# Genome-Wide Identification and Expression Analysis of Udp-Glucuronosyltransferases in the Whitefly Bemisia Tabaci (Gennadius) (HemipterA: Aleyrodidae)

**DOI:** 10.3390/ijms21228492

**Published:** 2020-11-11

**Authors:** Litao Guo, Wen Xie, Zezhong Yang, Jianping Xu, Youjun Zhang

**Affiliations:** 1 Institute of Bast Fiber Crops, Chinese Academy of Agricultural Sciences, Changsha 410205, China; guolitao@caas.cn; 2 Department of Plant Protection, Institute of Vegetables and Flowers, Chinese Academy of Agricultural Sciences, Beijing 100081, China; xiewen@caas.cn (W.X.); yyll88523@126.com (Z.Y.); 3 Department of Biology, McMaster University, Hamilton, ON L8S 4K1, Canada

**Keywords:** UDP-glucuronosyltransferases, *Bemisia tabaci*, adaptability, fecundity, RNAi

## Abstract

*Bemisia tabaci* (Gennadius) (Hemiptera: Aleyrodidae) is an important agricultural pest worldwide. Uridine diphosphate (UDP)-glucuronosyltransferases (UGTs) are one of the largest and most ubiquitous groups of proteins. Because of their role in detoxification, insect UGTs are attracting increasing attention. In this study, we identified and analyzed UGT genes in *B. tabaci* MEAM1 to investigate their potential roles in host adaptation and reproductive capacity. Based on phylogenetic and structural analyses, we identified 76 UGT genes in the *B. tabaci* MEAM1 genome. RNA-seq and real-time quantitative PCR (RT-qPCR) revealed differential expression patterns of these genes at different developmental stages and in association with four host plants (cabbage, cucumber, cotton and tomato). RNA interference results of selected UGTs showed that, when *UGT352A1*, *UGT352B1*, and *UGT354A1* were respectively silenced by feeding on dsRNA, the fecundity of *B. tabaci* MEAM1 was reduced, suggesting that the expressions of these three UGT genes in this species may be associated with host-related fecundity. Together, our results provide detailed UGTs data in *B.* *tabaci* and help guide future studies on the mechanisms of host adaptation by *B.* *tabaci*.

## 1. Introduction

Plants and insects are among the most common organisms on earth. Together, they make up approximately half of all known species of multicellular organisms. Plants and insects interact with each other in multiple ways, as mutualists, antagonists, or commensals. A very common form of interaction between insects and plants with significant agricultural and economic importance is insect herbivory on crop plants. In this interaction, insects feed on plants that can cause significant damage to the plants. However, over time, host plants may evolve resistance mechanisms by evolving morphological structures and/or biochemical pathways, e.g., synthesizing and secreting toxic substances, against insects. In turn, herbivorous insects may evolve specific counter mechanisms towards specific host plants. The result of such an arms race can lead to increased specialization of insects feeding only certain plants [1,2]. Alternatively, the insect may change its feeding behavior and expand its feeding range, evolving resistance mechanisms to feed on multiple plant species. Indeed, polyphagous insects are commonly found in nature. One example of a polyphagous insect is the two-spotted spider mite, *Tetranychus urticae* Koch (Acari: Tetranychidae), a generalist herbivore that feeds on many crop and ornamental plants. Indeed, *Tetranychus urticae* is a model species for understanding the adaptation mechanism of insects to host plants. Genome sequence and functional analyses have identified a variety of adaptation mechanisms in *T. urticae*, including expansion of genes coding for detoxification enzymes, such as cytochrome P450 monooxygenases, carboxyl-choline esterases, and glutathione-S-transferases, as well as a proliferation of cysteine peptidases, and MFS and ABC transporters [3,4,5,6]. In addition, *T. urticae* has a large repertoire of genes coding for uridine diphosphate (UDP)-glycosyltransferases (UGTs) and intradiol-ring cleavage dioxygenases [7,8,9,10,11]. Comparative analyses revealed that these genes in *T. urticae* were obtained through horizontal gene transfer but their expressions differed significantly depending on the specific host plants that they feed on [3,6,8,12,13,14].

UGTs are ubiquitous, found throughout all domains of life, in animals, plants, bacteria and fungi [15]. They catalyze the addition of UDP-sugars to small hydrophobic molecules, turning them into more water-soluble metabolites. In insects, UGTs play an important role in the regulation of endobiotics and in the detoxification of xenobiotics by catalyzing the conjugation of small lipophilic compounds with sugars to produce glycosides [15]. In addition, insect UGT enzymes are activated by a variety of plant allelochemicals [15,16]. Several studies have demonstrated that generalist insects can respond to specific secondary metabolites produced by different host plants by inducing changes in gene expression to enhance their fitness on a specific host plant [17,18,19,20,21]. In *Helicoverpa armigera*, *UGT41B3* and *UGT40D1* were found associated with detoxification of gossypol [16]. Snoeck et al. reported that multiple compounds of the flavonoid class of plant secondary metabolites were glycosylated by three UGTs (tetur02g09850, tetur22g00270 and tetur22g00440) in *T. urticae* [11]. In additional, the expression profiles of *T. urticae* UGT genes changed after host-plant shifts [22]. The expansion of UGTs in the Asian longhorned beetle *Anoplophora glabripennis*, another polyphagous insect, is also consistent with the role of UGTs in host adaptation [23].

*Bemisia tabaci* (Gennadius) (Hemiptera: Aleyrodidae), commonly known as the silverleaf whitefly or sweet-potato whitefly, is an important cosmopolitan agricultural pest that causes great damages to agricultural crops [24]. It’s been reported that *B. tabaci* can feed on 74 families and 500 species of host plants [24]. *B. tabaci* is a species complex, consisting at least 24 morphologically indistinguishable, closely related sibling species with different genetic and biological characteristics [25]. In many parts of the world, two of the species, *B. tabaci* MEAM1 (Middle East-Asia Minor 1) and *B. tabaci MED* (Mediterranean) are the most harmful and invasive [25,26,27]. They can damage plants both directly by feeding on phloem sap, excreting honeydew and indirectly by transmitting over 300 plant viruses [28,29]. In recent years, several studies have been conducted to investigate the interactions of *B. tabaci* and its host plants. The silverleaf whitefly has shown different preference and fitness on different host plants, for example, cotton, cabbage, pepper [30,31,32]. Its host preference has been found to be affected by host nutrition [33,34,35] and by specific host metabolites [34,36,37,38,39]. However, the underlying molecular mechanism for its differential preference and fitness is largely unexplored.

We have conducted a genome-wide investigation of the UGTs in *B. tabaci* MEAM1. Our overall goal was to understand the involvement of UGTs in the host adaptability in *B. tabaci*. We first identified the putative UGT genes from the *B. tabaci* MEAM1 genome and transcriptome databases. We described the phylogenetic and structural analyses of the *B. tabaci* MEAM1 UGTs. The expressions of these UGTs were assessed by transcriptomic analysis and real-time quantitative PCR (RT-qPCR) during its different developmental stages and on four host plants. We also verified the function of selected UGT genes in fecundity and survival rate by RNAi. Our results contribute to our understanding of the insect-host interaction and may suggest the potential molecular mechanisms of host plant adaptability in *B. tabaci*.

## 2. Results

### 2.1. Identification and Phylogenetic Analysis of B. tabaci MEAM1 UGT Genes

**Figure 1:**
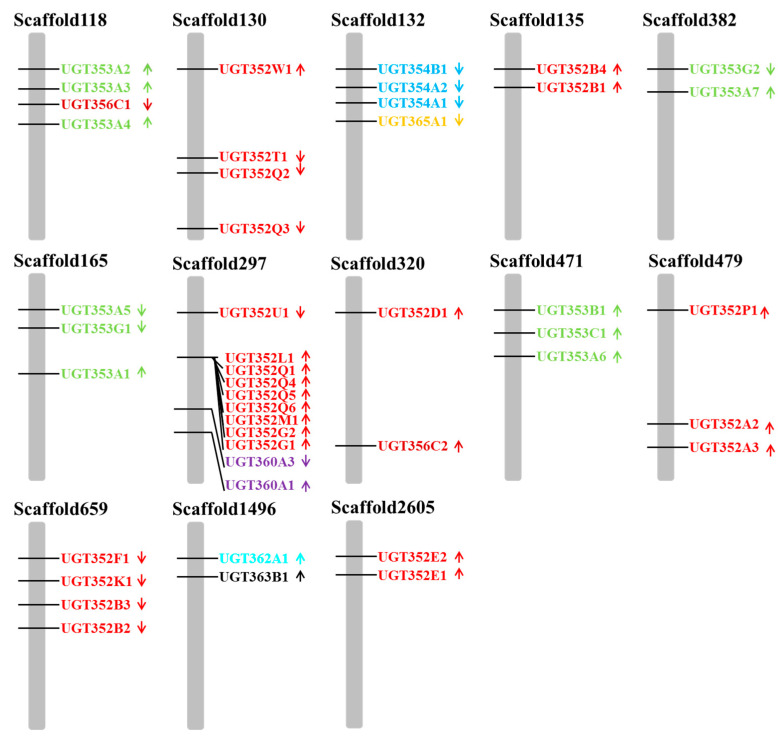
The relative physical positions of UGT genes on scaffolds of the *B. tabaci* MEAM1 genome. Arrows indicate the transcription direction of UGTs and genes in the same color belong to the same gene family. Accession numbers of UGT protein sequences used are listed in Table S1. Only scaffolds with more than 2 UGTs are shown in this Figure.

A total of 76 UGT genes were identified in the genome of *B. tabaci* MEAM1. The detailed information about each UGT gene is listed in Table S1. Following the current nomenclature guidelines of the UGT Nomenclature Committee, UGT genes investigated in this study were submitted to the UGT Nomenclature website (http://prime.vetmed.wsu.edu/resources/udpglucuronsyltransferasehomepage) to identify their current classifications. The identified names are shown in Table S1 [40]. In our study, the UGT gene annotations in the *B. tabaci* MEAM1 genome were changed due to greater availability of relevant data in databases over the original version by Chen et al. (2016) [41]. Five genes previously thought to be UGT’s (Bta01138, Bta02163, Bta07609, Bta09385, Bta09704) have been omitted from our list due to the revised concept of the gene family, while BtaUGT1 has been added to the list (Table S1). The 76 UGT genes in the *B. tabaci* MEAM1 genome are divided into 15 subfamilies (*UGT352*, *UGT353*, *UGT354*, *UGT355*, *UGT356*, *UGT357*, *UGT358*, *UGT359*, *UGT360*, *UGT361*, *UGT362*, *UGT363*, *UGT365*, *UGT366*, *UGT50*) (Table S1). Among these 76 UGT genes, 46 were distributed in thirteen scaffolds (Figure 1). Specifically, one scaffold (#297) contained eleven UGT genes (*UGT352G1*, *UGT352G2*, *UGT352L1*, *UGT352M1*, *UGT352Q1*, *UGT352Q4*, *UGT352Q5*, *UGT352Q6*, *UGT352U1*, *UGT360A1*, *UGT360A3*). Overall, the number of UGT genes in the *B. tabaci* MEAM1 genome is similar to several other phytophagous insects and phytophagous mites, for example, *Locusta migratoria* (68), *Acyrthosiphon pisum* (72) and *Tetranychus urticae* (81) (Table 1).

**Figure 2:**
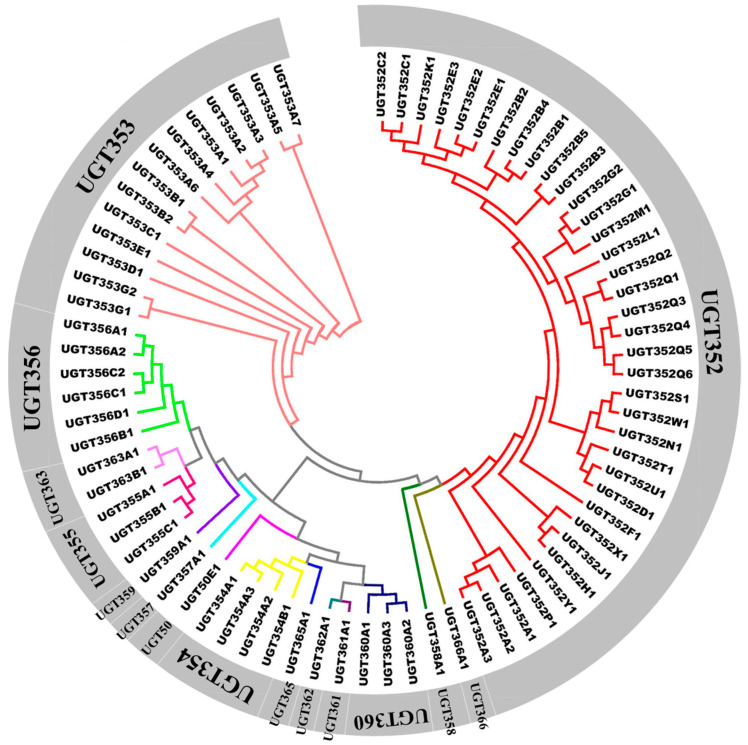
Phylogenetic relationships among the 76 UDP-glucuronosyltransferases (UGTs) in *B. tabaci* MEAM1. Amino acid sequences were aligned using ClustalW and subjected to a Neighbor-Joining (NJ) analysis by MEGA6 [42].

**Figure 3:**
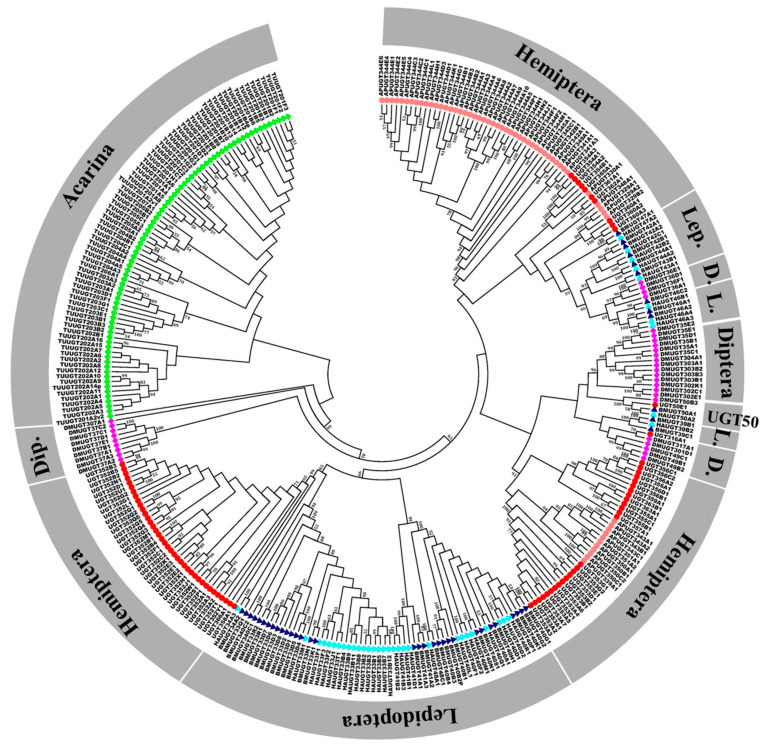
Phylogenetic relationships among UDP-glucuronosyltransferases (UGTs) in *B. tabaci* MEAM1 and other organisms. The amino acid sequences of full-length UGTs were aligned using ClustalW and subjected to a neighbor-joining (NJ) analysis by MEGA6 [42]. Numbers at the branch points of the nodes represent the values resulting from 1000 replications. Hemiptera: The dark red represents *Bemisia tabaci*, light red represents *Acyrthosiphon pisum* (Ap); Lepidoptera: the dark blue represents *Bombyx mori* (Bm), light blue represents *Helicoverpa armigera* (Ha); Diptera: the pink represents *Drosophila melanogaster* (Dm); Arachnoidea: the green represents *Tetranychus urticae* (Tu).

Phylogenetic analyses of the *B. tabaci* MEAM1 UGTs and UGTs from several other insects were constructed by the neighbor-joining (NJ) method using full-length UGT protein sequences (Figure 2 and Figure 3). In the phylogenetic tree of *B. tabaci* MEAM1 UGTs, the largest subfamily was *UGT352* containing 36 genes and accounting for 47.37% of all *B. tabaci* MEAM1 UGT members (Figure 2). The second most abundant subfamily was UGT353, which contained 14 genes in the *B. tabaci* MEAM1 genome. As expected, the UGT352 subfamily and UGT353 subfamily were clustered into their respectively groups (Figure 2). Among the species, the UGT families of different insects showed a variety of phylogenetic patterns; from conserved subfamilies with one member in each species clustered together like UGT50 (*DMUGT50B3*, *UGT50E1*, *BMUGT50A1*, *HAUGT50A2*) to large species-specific “blooms” such as UGT344 in aphid (*A. pisum*). Interestingly, the UGT genes in *B. tabaci* MEAM1 generally clustered together (Figure 3). In total, two phylogenetic subfamilies, *UGT352* and *UGT353* appeared to have expanded more than the others during the evolution of the UGT superfamily in *B. tabaci* MEAM1. UGT50 was the only subfamily found nearly universally distributed in the insects surveyed, composed of one member in each species except the pea aphid. In *B. tabaci* MEAM1, only one member of the UGT50 subfamily (*UGT50E1*) was found and it was more similar to UGT50 genes of other insects than to other UGTs from the *B. tabaci* MEAM1 genome.

### 2.2. Expression of the UGT Genes in Different Host Plants and Developmental Stages

**Figure 4:**
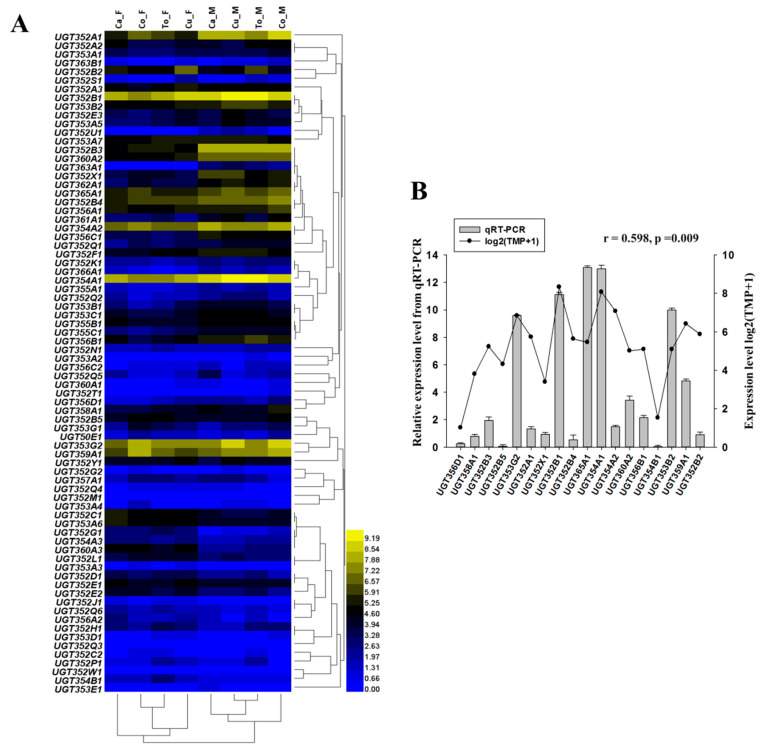
Expression profiling of UGT genes in MEAM1 was assessed using transcriptome data and RT-qPCR. (**A**) Expressions profiles of UGT genes in male and female adults that fed on four different host plants. The codes on the left are the gene ID numbers of the 76 UGTs in *B. tabaci* MEAM1. The mRNA levels, as represented by log2 (TPM + 1) values, are shown in the gradient heat map with colors ranging from blue (low expression) to yellow (high expression). Ca_F, females reared on cabbage; Ca_M, males reared on cabbage; Cu_F, females reared on cucumber; Cu_M, males reared on cucumber; Co_F, females reared on cotton; Co_M, males reared on cotton; To_F, females reared on tomato; To_M, males reared on tomato. (**B**) mRNA expressions of selected *B. tabaci* MEAM1 UGT genes by RT-qPCR. The bars represent expression level of *B. tabaci* MEAM1 UGT genes relative to the UGT352X. Data are presented as means ± SE. Spots indicated log2 (TMP + 1) values of UGT genes in Ca_F. Correlation between RNA-seq and RT-qPCR were tested using SPSS with Pearson and two-tailed test, *r*, Pearson correlation; *p*, significant.

The expression patterns of UGTs were investigated using RNA-seq datasets obtained from *B. tabaci* MEAM1 feeding on four different host plants. In whiteflies of different sexes or feeding on different host plants, the expressions of many UGT genes were different (Figure 4). For example, the expression of *UGT352B1* was the highest in females feeding on cabbage, cucumber, tomato and in males feeding on tomato, the expression of *UGT353G2* was the highest in females feeding on cotton, and the expression of *UGT354A1* was the highest in males feeding on cabbage, cotton and cucumber. The following seven UGTs, *UGT352A1*, *UGT352B1*, *UGT352B2*, *UGT353G2*, *UGT354A1*, *UGT354A2* and *UGT359A1*, had significantly different expressions in different host plants. Interestingly, five UGTs (*UGT352A1*, *UGT352B3*, *UGT352B4*, *UGT354A1* and *UGT360A2*) had different expressions between the female and male whiteflies. In all four female samples, two UGT genes *UGT353A3* and *UGT363A1* were not expressed. In contrast, in the four male samples, three UGT genes, *UGT352Q3*, *UGT352M1* and *UGT352W1* were not expressed. The expression levels of 37 UGT genes (*UGT352A1-3*, *UGT352B1-5*, *UGT352C1*, *UGT352E1-3*, *UGT352F1*, *UGT352L1*, *UGT352X1*, *UGT352Y1*, *UGT353A1*, *UGT353A5-7*, *UGT353B2*, *UGT353C1*, *UGT353G2*, *UGT354A1-2*, *UGT355B1*, *UGT355C1*, *UGT356A1*, *UGT356B1*, *UGT356C1*, *UGT358A1*, *UGT359A1*, *UGT360A2-3*, *UGT361A1*, *UGT362A1*, *UGT365A1*) showed more than twofold differences (Table S3) among different host plants.

**Figure 5:**
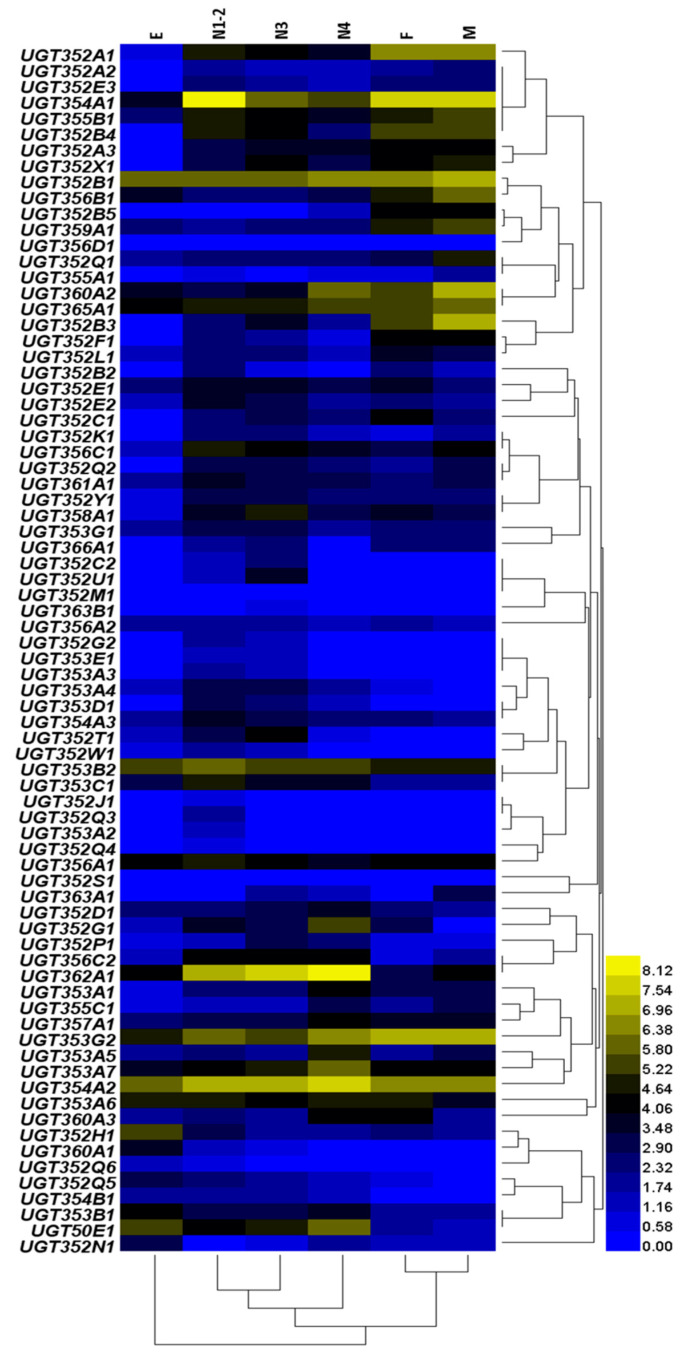
Gene expressions of UGTs across different developmental stages from MEAM1. The codes on the left are the gene ID numbers of the 76 UGTs in *B. tabaci* MEAM1. The mRNA levels, as represented by log2 (TPM + 1) values, are shown in the gradient heat map with colors ranging from blue (low expression) to yellow (high expression). E, egg; N1-2, 1st- and 2nd- instar nymphs; N3, 3rd- instar nymphs; N4, 4th- instar nymphs; F, adult female; M, adult male.

RNA-seq data were also used to investigate how the expression profiles of UGTs were affected by different developmental stages of *B. tabaci* MEAM1 (Figure 5). The results revealed that *UGT354A1* was the most highly expressed among the UGTs in the 1st-2nd instars (N1-2) and in the adults (F and M). In the 3rd instars (N3) and the 4th instars (N4), the expression of *UGT362A1* was the highest. In the eggs (E), the expression of *UGT352B1* was the highest. The expression levels of only 18 UGT genes (*UGT352B1*, *UGT352E1*, *UGT353A6*, *UGT353A7*, *UGT353B2*, *UGT353C1*, *UGT353G2*, *UGT354A1*, *UGT354A2*, *UGT354A3*, *UGT355B1*, *UGT356A1*, *UGT356B1*, *UGT357A1*, *UGT359A1*, *UGT360A2*, *UGT362A1*, *UGT365A1*) were more than two folds through all developmental stages (Table S3). Other notable features included: (i) *UGT363A1* and *UGT356D1* were not expressed in egg stage or the 1st- 2nd instars (N1-2); (ii) *UGT356D1* had the lowest expression in the 3rd instars (N3); (iii) *UGT353A2*, *UGT352J1* and *UGT352M1* were not expressed in the 4th instars (N4); (iv) *UGT352S1* and *UGT352M1* were not expressed in the female (F); and (v) *UGT352Q3*, *UGT360A1*, *UGT352W1* and *UGT352M1* were not expressed in the male (M). Most of UGT genes were expressed at very low levels through their developmental stages. Together, these results suggest that most UGTs are likely subject to fine-scale regulations.

### 2.3. mRNA Expressions of Selected UGT Genes by RT-qPCR

For the UGT genes showing high expression levels in the RNA-seq data, their expression profiles were further confirmed by RT-qPCR (Figure 4). 14 UGT genes (*UGT352A1*, *UGT352B1*, *UGT352B2*, *UGT352B3*, *UGT352B4*, *UGT352X1*, *UGT353B2*, *UGT353G2*, *UGT354A1*, *UGT354A2*, *UGT356B1*, *UGT359A1*, *UGT360A2*, *UGT365A1*) had distinct high expression (yellow) in *B. tabaci* MEAM1 feeding on different host plants, and UGT352B5, UGT358A1, UGT356D1, UGT354B1 had low expression (black and blue). Therefore, we selected these 18 UGT genes to verify the expression by RT-PCR. Among the 18 genes analyzed, *UGT354B1* had the highest expressional level, followed by *UGT352B5*, *UGT352B4* which was slightly different from the RNA-seq data. However, statistical analysis by SPSS 17.0 showed an overall significant correlation between the RNA-seq data and RT-qPCR data (r = 0.598, *p* = 0.009).

### 2.4. Effect of RNAi of UGTs on B. tabaci MEAM1 Adaptability

**Figure 6:**
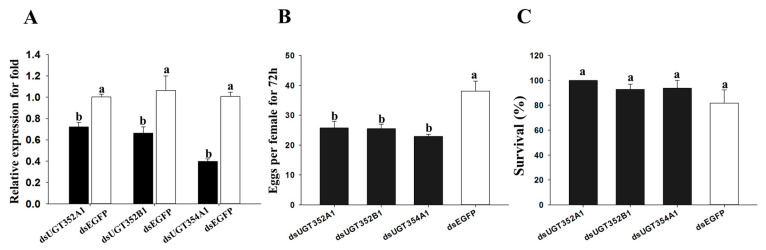
Silencing of UGT genes expression by oral feeding of dsRNA. (**A**) Suppression of UGT gene expression after *B. tabaci* MEAM1 adults were fed dsRNA for 48 h, with adults fed dsEGFP as a control. The expression of UGT genes was detected by RT-qPCR with EF1-α as the internal reference gene. (**B**) Effect of silencing UGT genes on *B. tabaci* MEAM1 fecundity (fecundity at 72 h per female after feeding on cabbage). (**C**) Effect of silencing UGT genes on *B. tabaci* MEAM1 survival (survival at 48h after feeding on dsRNA). Different letters above the bars indicate significant differences between treatments (*p* < 0.05; Tukey’s test; *n* = 4).

To investigate the potential functions of UGTs in *B. tabaci* MEAM1, three UGTs were cloned and dsRNAs of the three UGTs were synthesized and separately fed to *B. tabaci* MEAM1 adults. The expression levels of UGT352A1, UGT352B1 and UGT354A1 were significantly suppressed after feeding with the dsRNAs (Figure 6). After feeding on the dsRNAs for 48 h, the female adults were transferred to cabbage for 72 h. At the phenotype level, while the survival of the female adults was not significantly different from the negative control, the number of eggs that an individual female laid was significantly reduced compared to those fed with dsEGFP (Figure 6).

## 3. Discussion

In the present study, 76 UGT genes were identified in the *B. tabaci* MEAM1 genome (Table S1). This number of UGTs in *B. tabaci* MEAM1 is similar to number found in generalist insects (*Acyrthosiphon pisum*, 72 genes; *Locusta migratoria*, 68 genes) and mites (*Tetranychus urticae*, 81 genes) [41]. Consistent with previous results, the UGT genes in the polyphagous *B. tabaci* was more than those in the oligophagous insects feeding on plants, such as *Rhodnius prolixus* (16 genes) and *Apis mellifera* (11 genes) [38]. Table 1 summarized the number of UGT genes and feeding habits of 17 different insects (Table 1). We found that the number of UGT genes in non-phytophagous insects was generally low and in phytophagous insects, the number of UGT genes in polyphagous insects was higher than non-polyphagous insects (Table 1). These results are consistent with the hypothesis that the diversity of plant diet is a significant factor for UGT gene abundance in phytophagous insect genomes.

Our phylogenetic analysis identified a remarkable expansion of the *UGT352* and *UGT353* families in the *B. tabaci* MEAM1 genome. Our result was similar to the observed high number of genes in *UGT344* family in *Acyrthosiphon pisum*, *UGT201* family in *Tetranychus urticae*, and *UGT33* family in *Helicoverpa armigera* where each of them showed significant expansion in their respective genomes. Based on the cluster patterns in Figure 3, those clustered closely to each other likely have similar origins and functions. In the *B. tabaci* MEAM1 genome, the examples include *UGT316A1* and *DMUGT317A1* in one branch; *UGT50E1*, *BMUGT50A1*, *HAUGT50A2* and *DMUGT50B3* in one branch; *UGT361A1*, *UGT362A1* and *APUGT330A1* in one branch; and *UGT360A1*, *UGT360A2*, *UGT360A3*, *APUGT329A1*, *APUGT329A2* and *APUGT329B2* in one branch. Previous studies have shown that the generalist species *A. pisum*, *T. urticae* and *H. armigera*, are able to thrive on many hosts and that their UGTs showed differential expressions among the hosts. For example, UGT genes were suggested as playing important roles in the adaptation mechanism of *T. urticae* [22]. Similarly, the expansion of UGTs in *Anoplophora glabripennis* was thought to be related to its ability to feed on a broad range of host plants [23]. Therefore, we infer that the greatly expanded UGT genes in *B. tabaci* are at least partly related to its broad host range.

Insect UGT genes play diverse roles, including detoxification, digestion, olfaction and pigmentation and they are expressed in many different tissues [15,43]. The over-expressions of UGT genes in insecticide-resistant insects have been reported in *Leptinotarsa decemlineata* (Say), *Plutella xylostella* (L.), *Diaphorina citri Kuwayama*, *Tetranychus cinnabarinus* (Boisduval), *Anopheles sinensis* Wiedemann and *Aphis gossypii* Glover [43,44,45,46,47,48]. It has also been reported that UGTs were induced by insecticide exposures in *Spodoptera exigua* (Hübner) and *Bemisia tabaci* (Gennadius) [49,50]. However, detoxification by insect UGTs as a mechanism of adaptation to host plants has not been demonstrated. In our study, more UGT genes showed differential expression when fed with different host plants (37 UGT genes) than among different development stages with the same diet (18 UGT genes) (Table S3). Together, our results suggest that UGT gene expressions in *B. tabaci* are likely more related to host adaptation than to its development.

In general, insect adaptation to host plants is measured by comparing trait values (e.g., fecundity, survival, acceptance) [2]. To test whether the observed expression differences among UGTs were associated with adaptation to host plants, we selected three UGT genes (*UGT352A1*, *UGT352B1* and *UGT354A1*) and tested their knockdown effects on adult females through RNAi, using cabbage as the diet. Our results showed the fecundity of female *B. tabaci* MEAM1 was reduced in all three RNAi treatments, supporting their potential role in host adaptation of *B. tabaci* to cabbage. Our results are also consistent with previous research results on the role of UGTs in host adaptation in other phytophagous insects [17,18,19,20,21,22]. For example, nine UGT genes in three insects *(HAUGT41B3* and *HAUGT40D1 in H. armigera* [16]; *TUUGT204B2*, *TUUGT202A2*, and *TUUGT202A15* in *T. urticae* [11]; and *UGT330A3*, *UGT344D5*, *UGT348A3*, and *UGT349A3 in Myzus persicae*) [51] were associated with the detoxification of plant secondary metabolites, specifically gossypol, flavonoid and nicotine by the three insect species. As shown in Figure 3, *UGT354A1* was closely related to subfamilies of AP*UGT330A*, *APUGT344D*, AP*UGT348A*, and AP*UGT349A.* The knockdown expressions of three UGT genes (*UGT352A1*, *UGT352B1* and *UGT354A1*) by RNAi in *B. tabaci* MEAM1 reduced the its fecundity (Figure 6). The results suggest that *UGT354A1* expression likely contributed to *B. tabaci*’s tolerance and adaptation to secondary metabolites in cabbage. Interestingly, the *T. urticae* UGT genes showed a high degree of sequence similarity to those in bacteria [22]. Together with the lack of a signal peptide at the N-terminus and lack of the transmembrane domain at the C-terminus for the *T. urticae* UGT genes, the results suggested that the UGT genes in *T. urticae* were likely acquired from bacteria by horizontal gene transfer [22]. In our study, we found no robust evidence of horizontal gene transfer for any of the UGTs in the *B. tabaci* MEAMI genome. Specifically, none of the UGTs in the *B. tabaci* MEAMI genome was clustered with those in *T. urticae* nor with bacterial UGTs, consistent with those found by Chen et al. [41]. Indeed, 31 UGT genes, including *UGT352B1*, in *B. tabaci* MEAMI had no signal peptide in the N-terminus (Table S1), one of the criteria used for identifying eukaryotic genes of potential bacterial origin. Our results here provide foundations from which to further investigate the roles of specific UGTs in pest adaptation to additional host plants and potential mechanisms for their controls and managements.

## 4. Materials and Methods

### 4.1. Insect Strain

*B. tabaci* MEAM1 (Middle East- Asia Minor 1) were originally collected in 2004 from a field of cabbage, *Brassica oleracea* L. cv. Jingfeng 1, in Beijing, China [28]. The *B. tabaci* MEAM1 insects used in this study were reared on cabbage, in a glasshouse with natural light and controlled temperature (26 ± 2 °C). The method used for monitoring our experimental populations was the same as that described previously [52].

### 4.2. Identification of UGTs in B. tabaci and Other Insects

To identify the putative UGT genes in the *B. tabaci* MEAM1 genome, we searched the genome (http://www.whiteflygenomics.org) [41] and transcriptome [53] (SRP064690) databases of *B. tabaci* MEAM1. Specifically, first, we used the annotated UGT genes from *Drosophila melanogaster* and *Acyrthosiphon pisum* as queries for homolog searches in the *B. tabaci* MEAM1 genome. Second, the identified putative homologs were annotated by blasting against the non-redundant (NR) protein sequence database of GenBank (http:www.ncbi.nlm.nih.gov/) with the blastp program [54]. Finally, the identified UGTs were searched against *B. tabaci* transcriptome database [42] (SRP064690) using the tblastn program [54], with manual checking and confirmation for each gene. According to the current nomenclature guidelines of the UGT Nomenclature Committee, preliminary grouping was done using the program H-CD-HIT [55] at 60% and 40% sequence identity as cut off values, and preliminary family and subfamily names were assigned on this basis.

### 4.3. Phylogenetic Analysis of B. tabaci MEAM1 UGT Genes

To understand the evolutionary relationship among the UGT genes within *B. tabaci* MEAM1 and between these UGT genes and those in several other insects, we retrieved the UGT sequences of *Acyrthosiphon pisum* (Ap), *Bombyx mori* (Bm), *Helicoverpa armigera* (Ha), *Drosophila melanogaster* (Dm), *Tetranychus urticae* (Tu) using the annotations in previous reports [15,22,56]. All the selected UGT amino acid sequences were aligned using ClustalW, a module of MEGA 6 [42]. A phylogenetic tree was constructed based on the aligned UGT protein sequences using the neighbor-joining (NJ) method based on the Jones–Taylor–Thornton (JTT) model with a uniform substitution rate combined with pairwise deletion and 1000 bootstrap replicates.

### 4.4. Expression profiling of UGT Genes

Expression profiling of UGT genes in *B. tabaci* MEAM1 was assessed using transcriptome data of different developmental stages and four different host plants (cabbage, cucumber, cotton, and tomato) as described earlier [57]. The RNA-seq data and processing method were the same as our previous report [57]. The relative expression levels of UGT genes were also verified through RT-qPCR (ABI 7500 Real-Time PCR system (Applied Biosystems, Forster city CA, USA)). Total RNA was extracted using Trizol reagent according to the manufacturer’s instructions (Invitrogen, Carlsbad, CA, USA). RNA was quantified using a Nanodrop 2000 (Thermo Scientific, Wilmington, DE, USA) and its purity was checked on 1% agarose gel. Gene-specific primers for 18 UGT genes were designed and used in PCR reactions. Each RT-qPCR reaction (20 μL) contained 7 μL ddH2O, 10 μL of 2 × SuperReal PreMix Plus (TIANGEN, Beijing, China), 0.8 μL l0 μM of each specific primer, 1 µL first-strand cDNA template and 0.4 μL of 50 × ROX Reference Dye (TIANGEN). The RT-qPCR program consisted of an initial denaturation at 95 °C for 15 min followed by 40 cycles of denaturation at 95 °C for 15 s, annealing at 60 °C for 30 s and extension at 72 °C for 32 s. The translation elongation factor 1 alpha gene (EF-1α) (GenBank accession NO. EE600682) [58] was used as reference gene and relative gene expression was calculated using the 2^ΔΔCT^ method [59]. Four technical replicates and three biological replicates were used for each treatment. Sequences of all primers used in this study are listed in Supporting Information Table S2. All gene primer amplification efficiencies were between 95–105%.

### 4.5. RNA Interference

The expression of *UGT352B1* and *UGT354A1* was the highest in transcriptome data of different host plants and RT-PCR data, and these three genes expressions differed significantly among the host plants (See Results below). Therefore, three UGT genes, *UGT352A1*, *UGT352B1* and *UGT354A1*, were selected for RNAi. In our RNAi experiment, the dsRNA for enhanced green fluorescent protein (EGFP) was used as the negative control. The dsRNA primers of *UGT352A1*, *UGT352B1*, *UGT354A1 and EGFP* (GenBank: KC896843) are listed in Table S2. The dsRNAs were prepared according to the T7 RiboMAX Express RNAi system protocols (Promega, Madison, USA). The RNAi bioassay was performed by directly feeding dsRNA to *B. tabaci* adults in a feeding chamber for 48 h. A 0.20-mL drop of diet solution that contained 5% yeast extract and 30% sucrose (wt/vol) with 100 ng of dsRNA was placed in the chamber. The details of feeding were performed as described in a previous study [60]. Approximately 60 adults (mixed sexes) were used for RNAi treatment of each UGT gene, and each treatment repeated four times. The experiment was conducted in an environmental chamber at 26 °C, a photoperiod of L14: D10, and 80% RH. After 48 h, five females were randomly selected and transferred to cabbage to lay eggs for 72 h. The remaining whitefly adults were collected for analysis of the expression levels of targeted UGT genes. The survival rate of the female and the number of eggs that they laid were recorded.

### 4.6. Statistical Analysis

One-way ANOVA was used to compare the gene expression levels and survival rates among different treatments. Means were compared with Tukey’s tests at *p* < 0.05. SPSS version 17.0 (SPSS Inc., Chicago, IL, USA) was used for statistical analyses.

## Figures and Tables

**Table 1:** The number of UDP-glucuronosyltransferases genes in different insects and mites.

Species	Number ^a^	Food Type	Diet Breadth
*Bemisia tabaci*	76	Phytophagous	Polyphagous
*Tetranychus urticae*	81	Phytophagous	Polyphagous
*Acyrthosiphon pisum*	72	Phytophagous	Polyphagous
Locusta migratoria	68	Phytophagous	Polyphagous
Helicoverpa armigera	42	Phytophagous	Polyphagous
*Diaphorina citri*	37	Phytophagous	Oligophagous
*Rhodnius prolixus*	16	Phytophagous	Oligophagous
*Danaus plexippus*	35	Phytophagous	Oligophagous
*Apis mellifera*	11	Phytophagous	Oligophagous
*Nilaparvata lugens*	20	Phytophagous	Monophagous
*Bombyx mori*	38	Phytophagous	Monophagous
*Nasonia vitripennis*	22	Carnivorous	Monophagous
*Camponotus floridanus*	21	Omnivorous	Polyphagous
*Tribolium castaneum*	27	Omnivorous	Polyphagous
*Anopheles gambiae*	24	Sanguivorous	Oligophagous
*Pediculus humanus*	4	Sanguivorous	Oligophagous
*Drosophila melanogaster*	35	Saprophagous	Polyphagous

^a^ The number of UGT genes in different insects was obtained from reference Chen et al., 2016 [41].

## Supplementary Materials

Supplementary materials can be found at https://www.mdpi.com/1422-0067/21/22/8492/s1.

## Abbreviations

UDP	Uridine diphosphate
UGTs	Uridine diphosphate glucuronosyltransferases
EF-1α	The translation elongation factor 1 alpha gene
MEAM1	Middle East-Asia Minor 1
TPM	Transcripts Per Million
