# Supplementary material for: Genome-Wide Identification and Expression Analysis of Udp-Glucuronosyltransferases in the Whitefly Bemisia Tabaci (Gennadius) (HemipterA: Aleyrodidae)
Source: Int J Mol Sci. 2020 Nov 11;21(22):8492. doi: 10.3390/ijms21228492 (PMC7697561; doi:10.3390/ijms21228492)
Supplement: Supplementary file 1 [file ijms-21-08492-s001.pdf]

Table S1. UDP-glucuronosyltransferases (UGTs) identified in the *B. tabaci* MEAM1 genome

Gene ID	Gene name	Blast hit/Species	Scaffold	Strand	Identities/E value	CDS Length	Exon number	TM	SignalP
Bta05951	UGT352A1	KDR12464.1/Zootermopsis nevadensis	Scaffold226	+	35%/3e-86	1605	7	1	√
Bta11108	UGT352A2	XP_008472929.1/Diaphorina citri	Scaffold479	-	34%/3e-89	1614	6	1	√
Bta11109	UGT352A3	KDR12464.1/Zootermopsis nevadensis	Scaffold479	-	36%/7e-90	1611	6	2	\
Bta02726	UGT352B1	KDR12464.1/Zootermopsis nevadensis	Scaffold135	-	37%/8e-98	1587	6	1	\
Bta13758	UGT352B2	KDR12464.1/Zootermopsis nevadensis	Scaffold659	+	37%/2e-95	1629	6	1	\
Bta13757	UGT352B3	XP_008472929.1/Diaphorina citri	Scaffold659	+	35%/3e-99	1587	6	1	√
Bta02725	UGT352B4	KDR12464.1/Zootermopsis nevadensis	Scaffold135	-	38%/7e-104	1581	6	1	\
Bta12077	UGT352B5	XP_008472929.1/Diaphorina citri	Scaffold533	-	34%/4e-91	1590	6	1	√
Bta14671	UGT352C1	KDR12464.1/Zootermopsis nevadensis	Scaffold811	+	37%/3e-96	1590	6	1	√
Bta00822	UGT352C2	KDR12464.1/Zootermopsis nevadensis	Scaffold111	+	38%/6e-101	1560	6	1	\
Bta08294	UGT352D1	KDR12464.1/Zootermopsis nevadensis	Scaffold320	-	35%/5e-93	1584	6	2	\
Bta06665	UGT352E1	KDR23148.1/Zootermopsis nevadensis	Scaffold2605	-	36%/7e-83	1593	6	1	√
Bta06664	UGT352E2	KDR12464.1/Zootermopsis nevadensis	Scaffold2605	-	36%/8e-82	1476	6	1	\
Bta12440	UGT352E3	KDR12464.1/Zootermopsis nevadensis	Scaffold564	-	38%/3e-90	1593	6	1	√
Bta13755	UGT352F1	KDR12464.1/Zootermopsis nevadensis	Scaffold659	+	32%/2e-87	1584	6	2	\
Bta07610	UGT352G1	KDR12464.1/Zootermopsis nevadensis	Scaffold297	-	37%/4e-94	1617	6	2	\
Bta07608	UGT352G2	KDR12464.1/Zootermopsis nevadensis	Scaffold297	-	37%/2e-97	1602	4	1	√
Bta09701	UGT352H1	AGC84403.1/Locusta migratoria	Scaffold370000	+	35%/4e-91	1596	6	1	√
Bta03192	UGT352J1	AGC84403.1/Locusta migratoria	Scaffold147	+	33%/2e-85	1590	6	1	√
Bta13756	UGT352K1	KDR12464.1/Zootermopsis nevadensis	Scaffold659	+	36%/5e-89	1590	6	1	√
Bta07602	UGT352L1	KDR12464.1/Zootermopsis nevadensis	Scaffold297	-	35%/2e-84	1587	6	2	√

Bta07607	UGT352M1	AEW43116.1/Helicoverpa armigera	Scaffold297	-	33%/5e-85	1584	6	1	\
Bta12269	UGT352N1	KDR12464.1/Zootermopsis nevadensis	Scaffold562	+	37%/2e-93	1581	6	2	\
Bta11101	UGT352P1	AGC84403.1/Locusta migratoria	Scaffold479	-	35%/1e-80	1602	6	0	√
Bta07603	UGT352Q1	XP_008472929.1/Diaphorina citri	Scaffold297	-	35%/4e-94	1596	6	2	√
Bta02327	UGT352Q2	XP_008472929.1/Diaphorina citri	Scaffold130	+	34%/6e-89	1584	6	2	\
Bta02348	UGT352Q3	KDR12464.1/Zootermopsis nevadensis	Scaffold130	+	33%/2e-80	1527	6	1	√
Bta07604	UGT352Q4	KDR12464.1/Zootermopsis nevadensis	Scaffold297	-	34%/2e-80	1587	6	1	√
Bta07605	UGT352Q5	XP_022196004.1/Nilaparvata lugens	Scaffold297	-	37%/1e-90	1839	7	2	\
Bta07606	UGT352Q6	AEW43152.1/Bombyx mori	Scaffold297	-	34%/3e-88	1608	6	2	\
Bta01766	UGT352S1	KDR12464.1/Zootermopsis nevadensis	Scaffold123	-	37%/7e-72	1587	6	1	\
Bta02326	UGT352T1	KDR12464.1/Zootermopsis nevadensis	Scaffold130	+	35%/2e-92	1587	6	2	√
Bta07589	UGT352U1	XP_008472929.1/Diaphorina citri	Scaffold297	+	34%/7e-68	1575	6	1	\
Bta02306	UGT352W1	KDR12464.1/Zootermopsis nevadensis	Scaffold130	-	34%/2e-79	1569	6	1	√
Bta05604	UGT352X1	KDR12464.1/Zootermopsis nevadensis	Scaffold206	-	35%/1e-85	1590	7	1	√
Bta11864	UGT352Y1	KDR12464.1/Zootermopsis nevadensis	Scaffold52	+	26%/7e-48	1703	6	1	\
Bta04543	UGT353A1	XP_015369013.1/Diuraphis noxia	Scaffold165	-	36%/9e-99	1578	6	1	\
Bta01181	UGT353A2	KDR22771.1/Zootermopsis nevadensis	Scaffold118	-	34%/1e-96	1575	5	1	√
Bta01182	UGT353A3	KDR22771.1/Zootermopsis nevadensis	Scaffold118	-	36%/1e-94	1578	5	1	√
Bta01184	UGT353A4	XP_015369012.1/Diuraphis noxia	Scaffold118	-	35%/9e-96	1557	5	1	√
Bta04537	UGT353A5	XP_004525930.1/Ceratitis capitata	Scaffold165	+	33%/3e-96	1563	6	1	√
Bta10865	UGT353A6	XP_967924.1/Tribolium castaneum	Scaffold471	-	34%/7e-96	1539	7	1	√
Bta09948	UGT353A7	XP_015370076.1/Diuraphis noxia	Scaffold382	-	34%/4e-86	1548	6	1	√
Bta10863	UGT353B1	XP_015369012.1/Diuraphis noxia	Scaffold471	-	33%/1e-97	1563	5	2	√
Bta07368	UGT353B2	XP_015369012.1/Diuraphis noxia	Scaffold2816	+	35%/2e-99	1563	6	1	√
Bta10864	UGT353C1	XP_015371540.1/Diuraphis noxia	Scaffold471	-	35%/6e-82	1590	5	1	√

Bta15255	UGT353D1	XP_014289136.1/Halyomorpha halys	Scaffold923	-	34%/3e-88	1578	5	1	√
Bta09289	UGT353E1	XP_001949845.2/Acyrthosiphon pisum	Scaffold343	-	37%/1e-90	1551	5	2	√
Bta04539	UGT353G1	KDR11536.1/Zootermopsis nevadensis	Scaffold165	+	36%/5e-98	1545	5	1	√
Bta09947	UGT353G2	XP_015371540.1/Diuraphis noxia	Scaffold382	+	35%/7e-97	1560	6	1	√
Bta02603	UGT354A1	KDR09454.1/Zootermopsis nevadensis	Scaffold132	+	40%/7e-127	1554	6	1	√
Bta02602	UGT354A2	KDR09454.1/Zootermopsis nevadensis	Scaffold132	+	41%/3e-129	1539	6	1	√
Bta00718	UGT354A3	KDR09454.1/Zootermopsis nevadensis	Scaffold1098	-	41%/1e-124	1542	6	2	√
Bta02601	UGT354B1	KDR09454.1/Zootermopsis nevadensis	Scaffold132	+	39%/6e-121	1635	8	1	\
Bta09615	UGT355A1	XP_002427365.1/Pediculus humanus corporis	Scaffold3609	+	37%/5e-106	1581	6	2	\
Bta07348	UGT355B1	XP_002427365.1/Pediculus humanus corporis	Scaffold2808	+	40%/1e-112	1572	6	2	\
Bta00548	UGT355C1	XP_002427365.1/Pediculus humanus corporis	Scaffold109	-	39%/1e-114	1572	6	2	\
Bta08743	UGT356A1	KDR11536.1/Zootermopsis nevadensis	Scaffold325	-	41%/4e-124	1590	5	2	\
Bta02991	UGT356A2	KDR11536.1/Zootermopsis nevadensis	Scaffold14	-	39%/3e-111	1602	5	2	\
Bta01304	UGT356B1	XP_014289132.1/Halyomorpha halys	Scaffold1193	-	36%/3e-108	1569	5	1	√
Bta01183	UGT356C1	KDR11536.1/Zootermopsis nevadensis	Scaffold118	+	38%/1e-110	1560	5	2	\
Bta08372	UGT356C2	KDR11536.1/Zootermopsis nevadensis	Scaffold320	-	39%/3e-116	1551	5	1	\
BtaUGT1	UGT356D1	XP_022197631.1/Nilaparvata lugens	Scaffold835	-	41%/7e-122	1605	5	2	\
Bta04739	UGT357A1	KDR11536.1/Zootermopsis nevadensis	Scaffold1685	-	37%/1e-115	1605	5	1	√
Bta14119	UGT358A1	XP_008472929.1/Diaphorina citri	Scaffold73	-	33%/1e-73	1644	7	2	√
Bta07704	UGT359A1	KDR11536.1/Zootermopsis nevadensis	Scaffold299	+	36%/4e-111	1590	6	2	√
Bta07646	UGT360A1	XP_008191817.1/Tribolium castaneum	Scaffold297	-	44%/5e-133	1554	5	2	\
Bta02228	UGT360A2	KDR20744.1/Zootermopsis nevadensis	Scaffold1294	-	44%/3e-151	1560	5	1	√
Bta07623	UGT360A3	KDR20744.1/Zootermopsis nevadensis	Scaffold297	+	45%/2e-145	1557	5	1	√
Bta00300	UGT361A1	XP_008191789.1/Tribolium castaneum	Scaffold1040	+	41%/5e-132	1554	6	1	√
Bta03431	UGT362A1	KDR20744.1/Zootermopsis nevadensis	Scaffold1496	-	47%/1e-155	1716	7	2	\

Bta12684	UGT363A1	KDR11536.1/Zootermopsis nevadensis	Scaffold607	+	37%/1e-107	1605	6	1	√
Bta03440	UGT363B1	XP_014289134.1/Halyomorpha halys	Scaffold1496	-	36%/6e-101	1614	6	2	\
Bta02604	UGT365A1	KDR09454.1/Zootermopsis nevadensis	Scaffold132	+	42%/6e-140	1569	7	1	√
Bta07185	UGT366A1	AGC84403.1/Locusta migratoria	Scaffold2737	-	33%/1e-78	1545	7	1	√
Bta08213	UGT50E1	XP_008192323.1/Tribolium castaneum	Scaffold317	+	54%/0.0	1605	6	1	\

2

Table S2 Primers used for the RT-qPCR and RNA interference

Gene name	Forward primer sequence	reverse primer sequence	Application
UGT356D1	5′-CTCTGCTATTTCTATGTTGTTTG -3′	5′-TTGAGATTAGGGATTGGTTTC-3′	qRT-PCR-
UGT358A1	5′-ATCGCAATTCGTTCAATCTC-3′	5′-CCTCTATTATGATGGAGTCGTA-3′	qRT-PCR
UGT352B3	5′-TGTTCCTACAGTTGGTATCC-3′	5′-TGAAGTCACGAGATATTGCT-3′	qRT-PCR
UGT352B5	5′-GGTTGGTATTCCTTGGTTTG-3′	5′-GCCGCTTCATATTGTTGTAA-3′	qRT-PCR
UGT353G2	5′-GTCTCGCTTGTCTTTCTTAC-3′	5′-TGACTTCAACTCCTTATCCA-3′	qRT-PCR-
UGT352A1	5′- GTAGATGCAGGGATCGGAGC-3′	5′-CAGCACTCGCGGCTAAGATA-3′	qRT-PCR
UGT352X1	5′-GCTACTGAGGGTTACAAGAT-3′	5′-AACTACATTCGGACTGACAA-3′	qRT-PCR
UGT352B1	5′- AATTCTTGCCTTGTCAGAGA-3′	5′-CGTATGTGTAGTTATGGTTACTC-3′	qRT-PCR
UGT352B4	5′-ATCTCCGTTGTATTTCTGTCA-3′	5′-TTGCTATTATTCGCCATTCC-3′	qRT-PCR-
UGT365A1	5′-ATTGGTATCCCGTTGATGAT-3′	5′-AATTCTCCGAGGTAATGTCC-3′	qRT-PCR
UGT354A1	5′- ACTTCATGAGCGGAGCGAAA-3′	5′-TTGCGGAATTTGCCTGAACG-3′	qRT-PCR
UGT354A2	5′-CGGTTACGATGATGAATTGT-3′	5′-ATCCCTGTGAAACTTCTTGT-3′	qRT-PCR
UGT360A2	5′-AGAACTACCGAACAATCAGA-3′	5′-TGCTCTTCAATCTTGGAGTA-3′	qRT-PCR-
UGT356B1	5′-AGCCATAAGTTCAACATTCC-3′	5′-GTTATTGTATTCACCGTCCTT-3′	qRT-PCR
UGT354B1	5′-ACGACCCAGTAATACCTTAG-3′	5′-AGCAACATCAGCAATAATCA-3′	qRT-PCR
UGT353B2	5′-AACGCTTGGATTCATTACAG-3′	5′-GCAGTATTGGCAGTGAGA-3′	qRT-PCR
UGT359A1	5′-AGCATCAGTTCACTTACCAT-3′	5′-GGCACGATAGGAATACAAGA-3′	qRT-PCR
UGT352B2	5′-TTCAAGGAGAGGATGGAATC-3′	5′-GTAACTAGAGCGAGACTTATGT-3′	qRT-PCR
EF-1α	5′-TAGCCTTGTGCCAATTTCCG-3′	5′-CCTTCAGCATTACCGTCC-3′	qRT-PCR
UGT352A1	5′-GCGTGAAAGACTCACGATCA-3′	5′-CTTGTCATGAGGCGATGAGA-3′	Full length
UGT352B1	5′-CTCTCCGTGCAGTGAGCATA-3′	5′-AGTCAGGATTTATTGCGTTT-3′	Full length
UGT354A1	5′-CTCACGGCAGAAAGTAGCG-3′	5′-GCGTATCCAGCAGGAAGAA-3′	Full length

UGT354A1	5′-TAATACGACTCACTATAGGGAGAGCGTTGGCTACAGTCTCCTC-3′	5′-TAATACGACTCACTATAGGGAGACTCTGGAAATGGGGTACGAA-3′	RNAi
UGT352A1	5′-TAATACGACTCACTATAGGGAGATCAGAAATGGATACCCCAGC-3′	5′-TAATACGACTCACTATAGGGAGAGCTCGCCAAAGGAAGTACAG-3′	RNAi
UGT352B1	5′-TAATACGACTCACTATAGGGAGAGTCAACGGTGGTCCAAGACT-3′	5′-TAATACGACTCACTATAGGGAGATCAAGAAATTCTGTCGCACG-3′	RNAi
EGFP	5′-TAATACGACTCACTATAGGGAGACAGTGCTTCAGCCGCTAC-3′	5′-TAATACGACTCACTATAGGGAGAGTTCACCTTGATGCCGTTC-3′	RNAi

Table S3 Expression value of *UGTs* across different host and different developmental stages by RNA-seq data. The expression values were presented as log (TPM+1). Red data represent the >2 values in all different host, and blue data represent the >2 values in all different development stages.

Gene name	Ca_F	Co_F	Cu_F	To_F	Ca_M	Co_M	Cu_M	To_M	E	N1-2	N3	N4	F	M
UGT352A1	5.73	6.91	5.80	6.35	8.13	8.60	8.35	7.42	0.60	4.66	4.26	3.94	6.47	6.87
UGT352A2	4.67	3.34	4.48	3.48	4.03	4.63	3.70	5.05	0.07	2.24	1.39	1.38	2.17	2.65
UGT352A3	4.81	4.25	5.28	4.67	5.09	4.61	5.05	5.21	0.16	3.38	3.49	3.53	4.21	4.23
UGT352B1	8.33	7.75	9.11	8.14	8.56	8.59	9.37	9.85	6.34	6.31	6.17	6.42	6.76	7.11
UGT352B2	5.87	5.06	6.90	4.81	4.36	4.07	5.12	6.17	0.03	2.78	1.02	0.23	2.62	1.33
UGT352B3	5.23	5.50	5.18	5.65	7.99	8.05	8.33	8.36	0.14	2.78	3.74	1.97	5.22	7.04
UGT352B4	5.63	5.98	6.50	6.02	7.20	7.33	7.05	7.05	0.55	4.69	4.31	2.41	5.33	5.51
UGT352B5	4.31	5.23	4.36	4.73	4.25	5.18	4.08	4.38	0.04	0.24	0.29	1.22	4.24	4.15
UGT352C1	5.38	4.88	5.15	4.86	4.23	4.34	3.84	3.79	0.32	2.72	3.06	2.82	4.06	2.35
UGT352C2	0.23	0.75	0.53	1.00	0.22	0.17	0.52	0.87	0.07	1.35	2.42	0.15	0.14	0.30
UGT352D1	3.59	2.78	3.51	3.57	2.29	1.67	1.98	2.73	2.79	2.72	3.11	3.54	2.68	1.98

UGT352E1	4.97	4.31	5.06	4.58	4.15	4.01	4.08	4.26	2.66	4.04	3.87	3.05	3.94	2.87
UGT352E2	3.99	4.08	4.22	3.67	3.14	2.36	3.09	3.57	1.16	3.62	3.46	2.09	2.74	2.18
UGT352E3	3.61	3.18	4.38	3.89	3.76	3.72	4.77	4.58	0.14	2.59	1.80	1.45	2.36	2.53
UGT352F1	4.37	4.31	4.76	5.10	5.55	5.10	5.27	5.56	0.16	2.58	1.96	0.76	4.27	4.56
UGT352G1	2.96	3.13	3.26	3.42	0.00	1.74	0.95	1.00	1.39	3.56	3.18	5.74	3.26	0.26
UGT352G2	0.00	0.87	0.63	0.45	0.52	0.00	0.86	0.61	0.13	1.91	1.34	0.19	0.14	0.21
UGT352H1	2.98	2.18	3.22	3.40	1.63	2.68	1.70	2.77	5.28	3.25	1.93	2.26	2.59	1.84
UGT352J1	0.94	0.34	0.82	0.73	0.53	0.00	0.00	0.00	0.17	1.04	0.36	0.00	0.23	0.10
UGT352K1	2.16	2.14	2.04	1.42	2.96	2.75	2.41	3.13	0.12	2.79	2.44	1.47	1.15	2.16
UGT352L1	3.59	4.05	4.44	4.01	2.80	3.04	3.44	3.24	1.18	2.53	2.37	1.59	3.67	3.02
UGT352M1	0.00	0.05	0.00	0.00	0.00	0.00	0.00	0.00	0.03	0.10	0.35	0.00	0.00	0.00
UGT352N1	1.74	1.71	1.33	1.99	1.54	2.26	1.76	2.23	3.47	0.08	0.86	2.30	1.32	1.72
UGT352P1	1.07	1.17	1.50	2.05	1.00	0.00	0.73	1.99	0.69	1.45	2.97	2.50	0.58	0.86
UGT352Q1	1.98	3.40	3.39	3.18	3.82	4.44	4.16	4.18	1.97	2.88	2.32	2.79	3.23	4.71
UGT352Q2	2.37	0.74	1.48	1.81	2.33	1.78	3.21	2.82	0.09	3.24	3.17	2.87	1.97	2.99
UGT352Q3	0.23	0.00	0.62	0.52	0.00	0.00	0.00	0.00	0.11	1.76	0.30	0.06	0.40	0.00
UGT352Q4	0.57	0.96	0.71	0.72	1.24	0.00	0.69	0.65	0.54	0.93	0.49	0.30	0.25	0.39
UGT352Q5	2.08	0.91	1.64	0.82	3.34	2.22	1.08	1.72	3.03	2.38	2.07	1.55	0.62	0.54
UGT352Q6	2.13	1.50	1.65	1.99	1.74	0.97	0.76	1.33	1.29	0.95	0.34	0.56	0.38	0.41
UGT352S1	1.12	0.52	2.29	0.19	0.53	1.07	0.55	1.40	0.05	0.16	0.18	0.10	0.00	0.23
UGT352T1	0.00	0.00	0.44	0.65	0.31	0.96	0.00	0.00	1.26	3.23	4.27	0.71	0.22	0.12
UGT352U1	0.00	0.26	0.63	0.23	1.34	0.00	2.04	1.39	0.05	1.64	3.48	0.08	0.26	0.35
UGT352W1	0.00	0.00	0.00	0.22	0.00	0.00	0.00	0.00	0.88	1.79	1.54	0.07	0.08	0.00
UGT352X1	3.39	3.99	3.88	4.19	6.04	5.75	6.05	5.15	0.35	3.37	4.55	3.32	4.17	5.20
UGT352Y1	4.11	4.59	4.70	3.99	3.91	4.36	5.00	4.50	0.71	3.06	3.12	2.62	2.68	2.40
UGT353A1	3.58	3.14	3.76	3.03	3.37	4.04	3.30	3.88	0.63	2.80	2.71	4.51	2.96	3.14
UGT353A2	0.00	0.00	0.00	0.00	0.00	0.00	0.00	0.82	0.18	1.25	0.57	0.00	0.09	0.14

UGT353A3	0.25	0.68	0.80	0.48	0.10	0.00	0.00	0.18	0.36	1.92	1.63	0.04	0.16	0.05
UGT353A4	0.50	1.43	0.29	0.62	0.17	0.00	0.92	0.74	1.28	3.45	3.15	1.74	0.66	0.36
UGT353A5	3.18	3.23	4.16	3.79	3.74	4.26	4.68	4.51	1.75	2.50	2.20	5.18	2.30	2.99
UGT353A6	5.36	4.84	5.08	5.03	4.38	4.43	4.12	4.11	4.87	4.80	4.57	4.87	5.09	3.90
UGT353A7	4.81	4.95	5.44	5.27	5.24	4.78	5.42	5.52	3.84	4.13	4.76	5.89	4.23	4.44
UGT353B1	3.09	1.65	3.68	2.47	4.30	3.38	4.11	4.23	4.44	3.14	3.43	4.03	1.83	1.99
UGT353B2	5.09	5.04	5.45	4.86	5.28	5.38	6.09	6.06	5.36	6.09	5.52	5.36	4.80	4.95
UGT353C1	4.07	3.38	4.32	3.35	4.70	4.05	4.95	4.70	2.91	5.19	3.86	3.68	2.09	2.02
UGT353D1	0.61	0.17	1.20	1.14	0.00	0.85	0.00	0.49	0.35	3.15	2.79	1.67	0.46	0.48
UGT353E1	0.42	0.00	0.26	0.47	0.74	0.00	0.00	0.00	0.07	1.66	1.41	0.21	0.32	0.10
UGT353G1	2.53	4.12	3.03	3.43	1.91	3.91	3.10	2.91	2.10	2.98	3.17	1.84	2.49	2.84
UGT353G2	6.84	8.21	7.49	7.85	7.37	8.59	8.74	7.50	4.67	6.33	5.45	6.78	7.04	6.98
UGT354A1	8.07	7.84	8.17	7.24	8.93	8.97	9.44	9.73	3.55	8.21	6.29	5.72	7.72	7.92
UGT354A2	7.07	7.31	6.80	6.79	8.19	8.09	7.64	7.61	6.17	7.42	7.12	7.85	6.71	6.85
UGT354A3	3.18	2.79	3.06	2.60	1.67	2.25	1.77	2.12	2.18	3.64	3.40	2.67	2.56	2.31
UGT354B1	1.53	1.84	1.39	2.65	1.26	0.80	1.19	1.09	2.16	1.86	2.20	1.24	0.52	0.55
UGT355A1	1.62	0.93	1.47	0.89	2.23	1.43	1.42	2.35	0.09	0.58	0.31	0.94	1.13	1.98
UGT355B1	4.61	4.23	4.42	4.50	5.10	4.86	5.14	5.00	2.71	5.15	4.56	3.89	4.89	5.32
UGT355C1	3.13	2.44	3.33	2.91	4.57	4.09	4.09	4.35	0.81	1.69	1.70	3.32	2.21	3.00
UGT356A1	5.28	5.23	5.44	5.10	5.65	6.02	5.83	5.71	4.41	4.86	4.39	3.86	4.23	4.10
UGT356A2	3.17	1.57	2.14	1.62	1.82	0.00	0.58	1.91	1.92	1.79	2.03	1.35	1.79	1.28
UGT356B1	5.09	3.47	4.95	4.48	5.73	5.35	5.40	6.03	3.93	2.85	2.80	3.17	5.09	6.01
UGT356C1	3.87	3.76	3.56	3.21	5.31	5.22	4.78	4.81	1.26	5.17	4.41	3.73	3.02	4.07
UGT356C2	0.00	0.80	0.69	1.08	1.31	1.52	0.43	1.96	1.50	4.59	4.13	4.28	1.11	1.78
UGT356D1	1.01	0.67	2.24	1.36	2.08	3.13	2.13	1.56	0.00	0.00	0.13	0.19	0.37	0.39
UGT357A1	1.79	3.20	2.51	3.23	2.68	0.90	2.90	2.59	2.53	3.06	3.16	4.61	3.89	3.74
UGT358A1	3.80	3.87	4.14	4.43	5.01	5.40	4.42	4.27	1.03	4.04	4.75	3.47	3.54	3.26

UGT359A1	6.42	8.01	6.47	7.18	6.66	8.36	7.82	7.78	2.36	2.12	2.54	2.39	4.69	5.59
UGT360A1	0.43	0.37	0.69	0.00	0.70	1.10	0.00	0.00	3.67	1.67	1.06	0.29	0.38	0.00
UGT360A2	5.01	5.20	5.56	5.10	6.85	6.85	7.12	6.99	3.94	3.18	3.88	6.19	5.75	7.15
UGT360A3	4.85	4.94	4.97	4.59	2.52	3.01	2.27	2.80	2.25	2.33	1.78	4.21	4.32	2.25
UGT361A1	2.93	3.04	2.45	3.35	4.17	5.08	4.68	3.84	1.78	3.60	3.47	3.30	2.79	3.36
UGT362A1	3.09	4.59	3.57	3.68	5.07	5.57	5.90	5.05	4.45	7.21	7.67	8.70	3.37	4.41
UGT363A1	0.00	0.00	0.00	0.00	2.75	2.03	3.69	3.25	0.00	0.00	2.07	1.66	0.11	3.39
UGT363B1	0.76	0.21	0.77	0.63	0.35	1.36	0.88	0.82	0.11	0.14	1.15	0.20	0.23	0.17
UGT365A1	5.46	5.95	5.60	5.53	6.13	6.65	6.73	6.37	4.63	4.67	4.65	5.69	5.53	6.14
UGT366A1	1.31	1.23	1.24	0.57	2.36	1.49	1.90	2.21	0.24	2.12	2.68	0.50	2.49	2.32
UGT50E1	1.21	2.44	1.73	1.39	1.16	1.99	1.66	0.96	5.70	4.32	4.93	5.90	2.26	1.45
